# Acute Compartment Syndrome Following Reduction of Simple Elbow Dislocation

**DOI:** 10.7759/cureus.63145

**Published:** 2024-06-25

**Authors:** Kodai Maki, Tatsuhiko Muraoka, Yoshinori Ueno, Manabu Yamashita, Koji Yonemori

**Affiliations:** 1 Orthopaedic Surgery, Department of Orthopaedic Surgery, Yonemori Hospital, Social Medical Corporation Ryokusenkai, Kagoshima, JPN; 2 Orthopaedic Surgery, Department of Trauma Reconstruction Center, Yonemori Hospital, Social Medical Corporation Ryokusenkai, Kagoshima, JPN

**Keywords:** dislocation, elbow, elbow dislocation complication, compartment syndrome, acute compartment syndrome, upper extremity, forearm, simple elbow dislocation

## Abstract

Although rare, acute compartment syndrome may develop as a simple elbow dislocation after reduction without initial motor, sensory, or peripheral circulatory abnormalities. This report describes a rare case of this condition. Acute compartment syndrome remains a potential complication, even in a simple elbow dislocation without apparent initial abnormalities, and should be explained to patients. A peripheral nerve block during reduction may mask symptoms and delay recognition of acute compartment syndrome. This case highlights the importance of vigilant monitoring for acute compartment syndrome following reduction of simple elbow dislocations, especially when a peripheral nerve block is used during reduction.

## Introduction

In the United States of America, approximately 83,996 simple elbow dislocations occurred between 2011 and 2020, with an incidence rate of 2.98/100,000 person-years [1]. Patients with simple elbow dislocation should undergo radiographic assessment for reduction, be immobilized for 7-10 days, and start early range of motion exercises afterward [2]. Peripheral nerve blocks may be used during reduction to improve reduction feasibility and alleviate pain [3]. Acute compartment syndrome (ACS) occurs when there is elevated tissue fluid pressure within a closed osseofascial compartment, such as the forearm, causing insufficient capillary perfusion to sustain tissue viability [4]. Ischemia from ACS can cause myonecrosis within three hours; therefore, prompt surgical decompression by extensive fasciotomy is required if ACS is suspected [4,5]. We report a case of simple elbow dislocation with ACS following reduction under a supraclavicular brachial plexus block, although there were no neurological or peripheral circulatory deficits at the first medical examination.

## Case presentation

**Figure 1:**
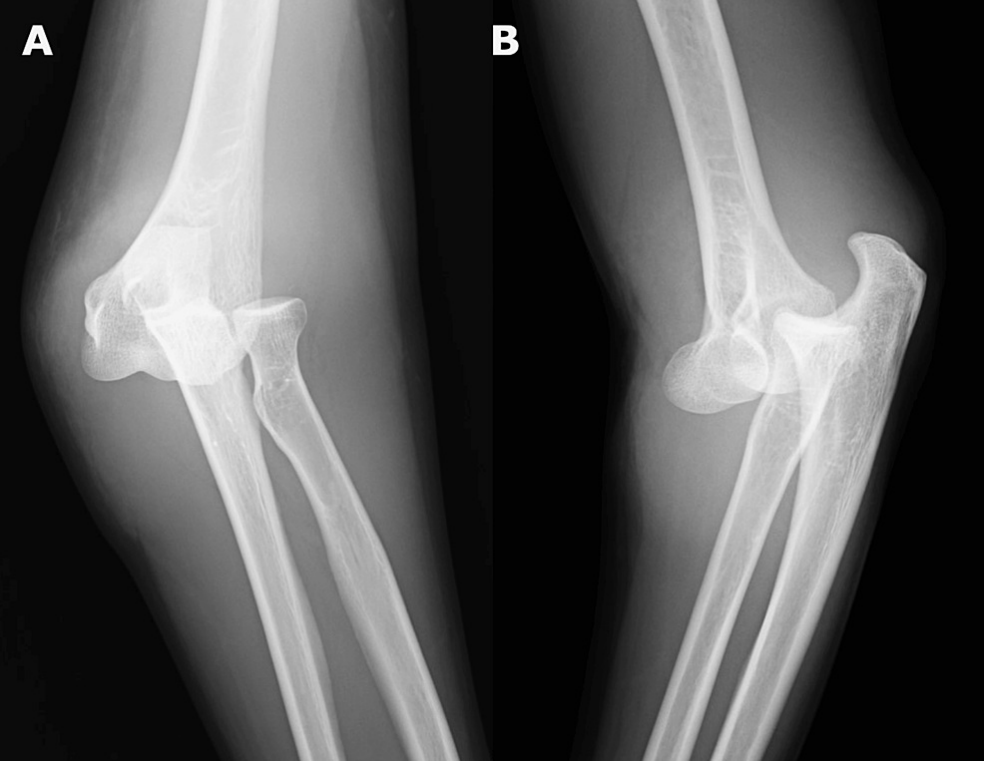
Elbow radiographs showing simple elbow dislocation before reduction, with no signs of bone fracture. (A) Anteroposterior view, (B) Lateral view

The patient was a 40-year-old woman with a history of hypertension who was not taking any medication. She was standing on a folding chair when she fell, was caught in the chair, and injured her left elbow. The left elbow was swollen, but there was no wound, bleeding, problems with hand motion or sensation, a capital refilling time (CRT) of less than two seconds, and the radial artery was palpable. Computed tomography (CT) and other investigations revealed no fractures (Figure 1), and the patient was diagnosed with simple elbow dislocation.

**Figure 2:**
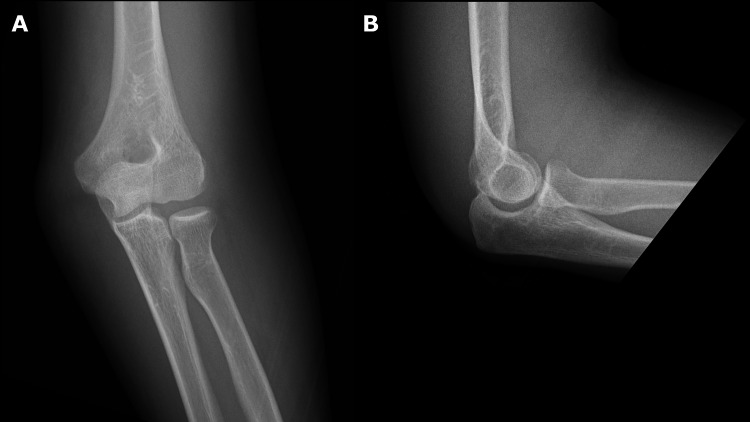
Elbow radiographs after closed reduction. (A) Anteroposterior view, (B) Lateral view

The swelling was severe, making manual reduction difficult. Therefore, fluoroscopic closed manual reduction was performed one hour after the injury under ultrasound-guided supraclavicular brachial plexus block with 20 cc of 1% mepivacaine (Figure 2). No resistance was observed during the reduction. After the reduction, the left elbow joint was stable, and the radial artery was palpable.

**Figure 3:**
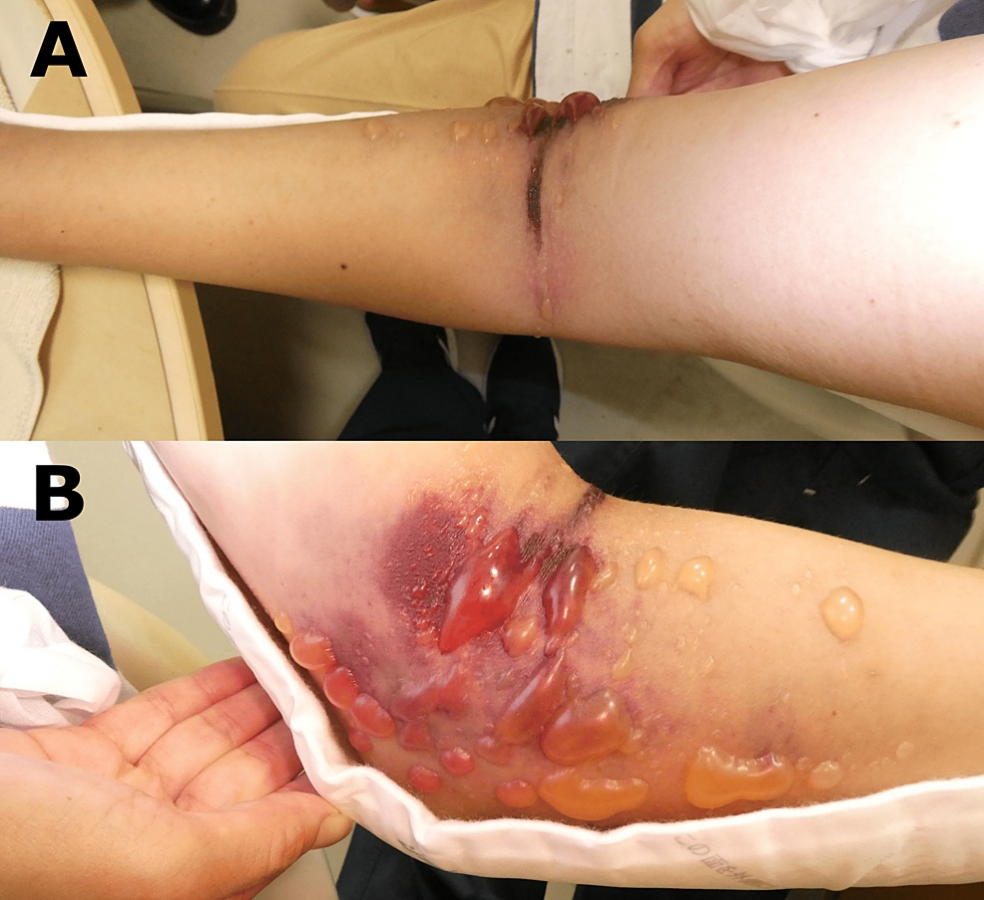
Blister formation on the medial side of the elbow. (A) Anterior view, (B) Medial view

**Figure 4:**
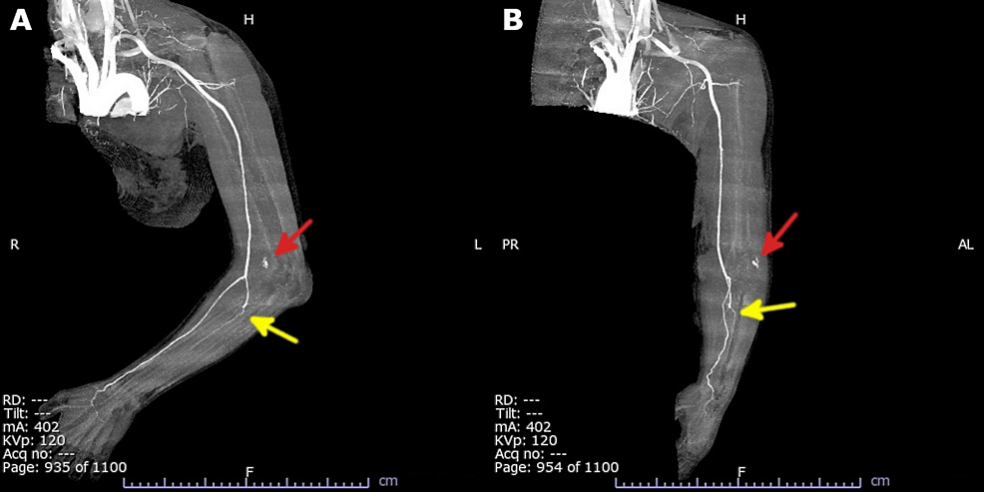
Contrast-enhanced CT image in the arterial phase showing contrast leakage from the brachial artery bifurcation (red arrow), poor ulnar artery delineation (yellow arrow), and no arterial rupture. (A) Lateral view, (B) Anteroposterior view

The patient was sent home with a Sheene fixation from the dorsum of the left hand to the left humerus and a plan for outpatient follow-up later. After returning home, the patient experienced severe swelling and pain in the forearm, blister formation on the medial side of the elbow, and numbness of the fingers. The patient contacted the hospital four hours after the injury and was re-examined five hours later after the injury. At the time of re-examination, blistering occurred mainly on the medial side of the elbow, but the radial artery was palpable, and the CRT was less than two seconds (Figure 3). However, 30 minutes after re-examination, the radial artery became poorly palpable, and ACS was diagnosed. Immediate contrast-enhanced CT revealed contrast leakage from the brachial artery bifurcation and poor delineation of the ulnar artery; however, no vascular rupture was observed (Figure 4).

**Figure 5:**
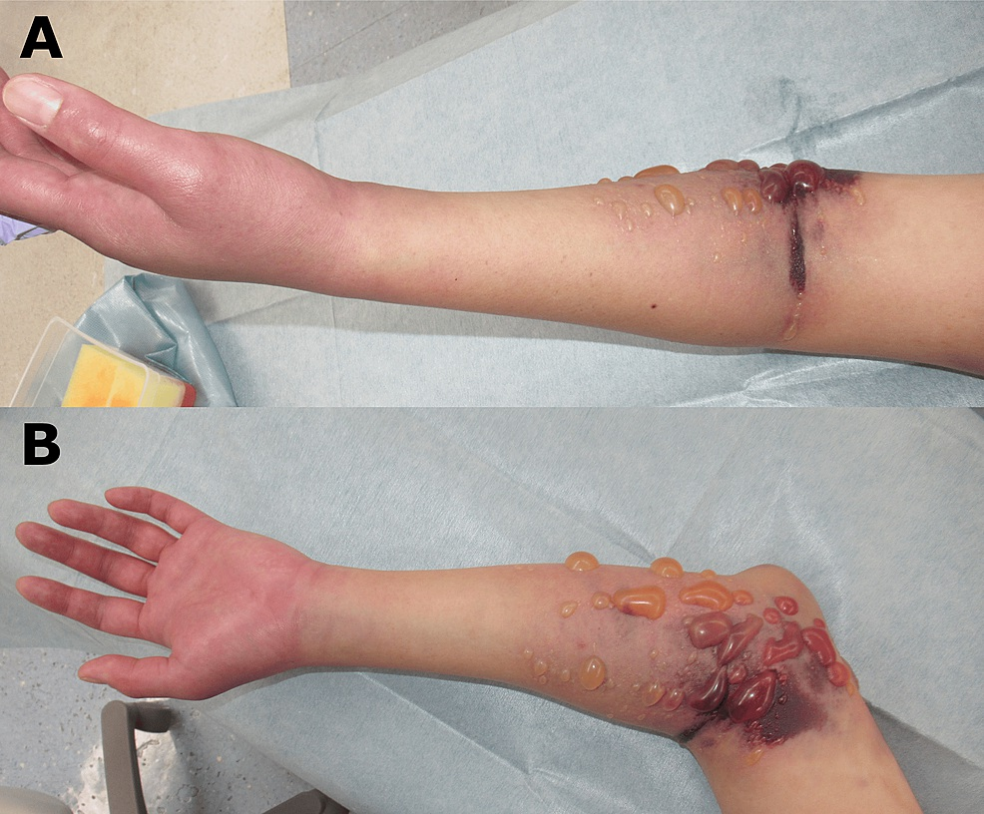
Blister exacerbation and poor hand coloration immediately before surgery. (A) Anterior view, (B) Medial view

**Figure 6:**
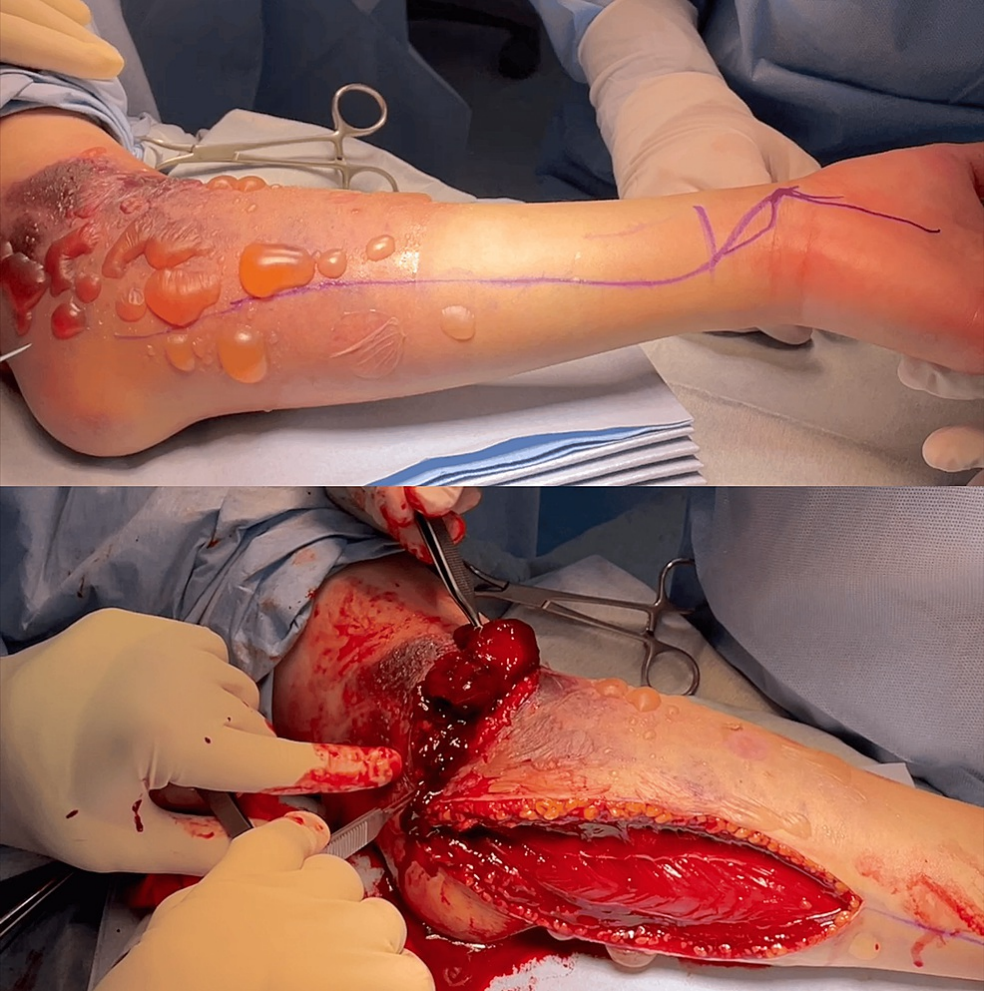
Intraoperative image of fasciotomy of the left forearm. Large hematomas were obvious after the forearm incision.

**Figure 7:**
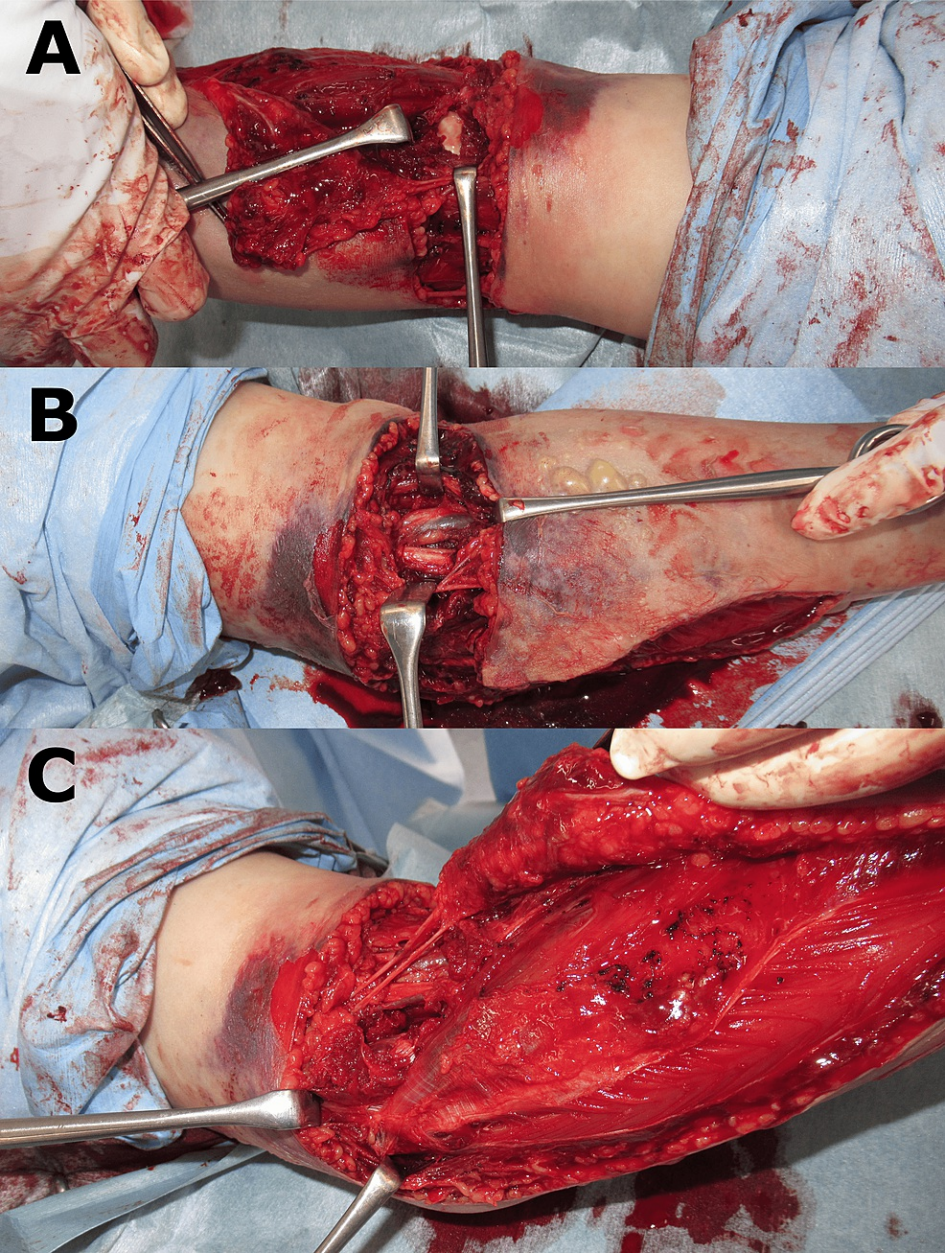
Intraoperative image of fasciotomy of the left forearm. (A) The anterior joint capsule of the elbow joint was detached, (B) No vascular rupture and nerve damage, (C) No flexor muscle group injury

The condition was determined to be urgent, and a fasciotomy was performed immediately without measuring pressure in the forearm compartment. Immediately before the surgery, the patient experienced blister exacerbation and poor hand coloration (Figure 5). After the forearm incision, fascial tension and mild muscle bulging were observed (Video 1). Large hematomas were also observed (Figure 6). Vascular rupture, nerve injury, or muscle injury was not observed, although the anterior joint capsule of the elbow joint was detached under direct visualization (Figure 7). A wet dressing was applied after confirming that the muscle had softened. Pressure in the three compartments of the forearm before closing the wound was 21 mmHg dorsally, 18 mmHg palmarly, and 31 mmHg in the mobile wad compartment. The wound was progressively closed in four stages: on the day of the first surgery and two, four, and eight days after the first surgery.

**Video 1:** Intraoperative video of fasciotomy of the left forearm. Fascial tension was evident.

After the first surgery, the forearm swelling was reduced, the radial artery was palpable, and there were no apparent differences in color between the right and left upper arms, forearms, and hands. The patient was discharged 16 days after the first surgery with no other neurological symptoms or significant loss of range of motion or muscle strength, although there was some sensory loss at the tip of the palmar side of the thumb. After discharge, the patient had no symptoms and was in good health.

## Discussion

This case report demonstrates two essential points. First, even after reduction, simple elbow dislocation without initial motor, sensory, or peripheral circulatory deficits may lead to ACS. Second, peripheral nerve blocks may delay the detection of ACS. 

A previous case report described ACS following simple elbow dislocation after reduction [6]. However, that report lacked comprehensive evaluations, including preoperative contrast-enhanced CT imaging and intraoperative vascular and nerve status assessments, which have failed to elucidate the mechanisms underlying ACS development.

The present case provides a more comprehensive evaluation, including contrast-enhanced CT findings and intraoperative observations, shedding light on possible pathogenesis. In this case, blunt trauma associated with simple elbow dislocation appeared to have caused vascular injury to small branches of the brachial artery and resultant hemorrhages, which likely led to the development of ACS. ACS secondary to a vascular injury is relatively rare [7]. Among the 106 cases of simple elbow dislocation without fracture reported at our hospital over the past 10 years (2014-2023), this was the only case that developed ACS, underscoring the rarity of this complication.

Intraoperatively, the brachial artery did not rupture, but large hematomas and surrounding hemorrhages were observed. In conjunction with contrast-enhanced CT findings, hematomas, and hemorrhages were considered to originate from small branches of the brachial artery. It was assumed that the ulnar artery was poorly visualized on contrast-enhanced CT because the hematoma formation compressed the ulnar artery. We concluded that blood leakage and hematoma formation due to vascular injury of the brachial artery branches associated with simple elbow dislocation increased pressure in the forearm compartment caused ACS.

In this case, the patient underwent fluoroscopy-guided closed reduction under supraclavicular brachial plexus block using 20 cc of 1% mepivacaine one hour after the injury. Four hours after the injury, the patient experienced severe swelling of the forearm, blister formation on the medial side of the elbow, and numbness of the fingers. Fasciotomy and desmotomy were performed six hours after the injury. The patient had an uneventful post-treatment course. Considering the patient's satisfactory clinical progress, the decision to administer the ACS treatment at that time was deemed appropriate. However, without peripheral nerve block, pain and numbness of the fingers may have been noticed before severe swelling and blistering developed, leading to earlier detection of ACS. Therefore, we concluded that in the present case, peripheral nerve block delayed the detection of ACS.

The possibility of ACS following simple elbow dislocation should be considered, even after reduction, as in this case. Given the potential for delayed detection of ACS, peripheral nerve blocks should be used cautiously during reduction. 

The classic signs of compartment syndrome, known as the "five Ps" (pain, pallor, paresthesia, paralysis, and pulselessness) [8], as well as other symptoms, should be explained to the patient and family. They should also be instructed to seek immediate medical attention if such signs develop. Re-examinations at shorter intervals are recommended as they may overlook significant findings. Follow-up visits should include an interview, physical examination with the removal of Sheene fixation, and, if necessary, quantitative ACS diagnostic tests such as Stryker needle, near-infrared spectroscopy (NIRS), ultrasound, and laboratory findings (creatine phosphokinase, creatinine, and urine myoglobin) should be performed [9].

## Conclusions

Simple elbow dislocation without initial motor, sensory, or peripheral circulatory abnormalities may lead to ACS. Patients with simple elbow dislocation should be fully informed about the risk of ACS. Peripheral nerve blocks during reduction should be performed cautiously because masking symptoms may delay the detection of ACS. Educating patients and families about ACS and performing frequent re-evaluations are critical for early recognition.
